# The household economic burden of human-only and zoonotic malaria, compared to other causes of acute febrile illness in Indonesia

**DOI:** 10.1136/bmjgh-2025-020504

**Published:** 2026-03-26

**Authors:** Patrick Abraham, Inke Nadia Diniyanti Lubis, Rintis Novivanti, Leily Trianty, Fahira Ainun Nisa, Pinkan Kariodimedjo, Ristya Amalia, Raden Andika Dwi Cahyadi, Tengku Mossadeq Al Qorny, Yudha Achmad Perlambang, Ranti Permatasari, Henry Surendra, Nicholas M Anstey, Matthew J Grigg, Angela Devine

**Affiliations:** 1 Centre for Health Policy, University of Melbourne, Melbourne, Victoria, Australia; 2 Department of Pediatrics, Faculty of Medicine Universitas Sumatera Utara, Medan, Indonesia; 3 Global and Tropical Health Division, Menzies School of Health Research, Charles Darwin University, Darwin, Northern Territory, Australia; 4 Eijkman Research Center for Molecular Biology, Jakarta, Indonesia; 5 Exeins Health Initiative, South Jakarta, Special Capital Region of Jakarta, Indonesia; 6 Faculty of Medicine Universitas Sumatera Utara, Medan, Indonesia; 7 Universitas Indonesia, Depok, Indonesia; 8 Infectious and Tropical Diseases Epidemiology, Public Health Program, Monash University Indonesia, Tangerang, Indonesia; 9 Oxford University Clinical Research Unit Indonesia, Faculty of Medicine, Universitas Indonesia, Jakarta, Indonesia; 10 Institute for Health Research, The University of Notre Dame Australia, Fremantle, Western Australia, Australia

**Keywords:** Malaria, Health economics, Global Health

## Abstract

Zoonotic malaria caused by infection with the monkey parasite *Plasmodium knowlesi* has emerged across Southeast Asia, particularly in areas previously close to elimination of non-zoonotic malaria. In Indonesia, some rural and remote areas must now consider strategies which target various *Plasmodium* species in hard-to-reach populations. Indonesia has mostly subsidised care at local health clinics for patients with malaria and other febrile illnesses; however, patients still face out-of-pocket costs. This study estimated household cost of illness due to malaria and non-malarial febrile illness in North Kalimantan and North Sumatra, Indonesia.

Household costs were estimated from individual patients as part of health facility-based cross-sectional surveys in eight health clinics across North Sumatra and North Kalimantan between January 2022 and October 2023. Direct costs due to medical and travel expenses, and indirect costs resulting from productivity losses were included. Overall, 2244 patients were recruited, including 153 (6.8%) malaria-confirmed cases. Five *Plasmodium* species were identified using validated PCR conducted on all participants: *P. vivax* (n=97), *P. knowlesi* (n=35), *P. malariae* (n=12), *P. falciparum* (n=3) and *P. ovale* (n=1), in addition to five mixed infections. Costs were inflated to 2023 Indonesian Rupiah and reported in US dollars (US$). A mean total cost of US$33 (SD=57) was reported for malaria episodes and US$17 for non-malarial fever episodes (SD=38), primarily composed of indirect productivity losses from time away from usual activities (70% and 61% of total cost for patients with malaria and other febrile illnesses, respectively). Overall, 16% of patients with malaria and 11% of patients with other febrile illnesses experienced catastrophic health expenditure from their illness episode.

Despite a largely subsidised health system, patients and families face other medical, travel and indirect costs when seeking care for febrile illnesses. These costs need consideration when designing malaria control policies, particularly in near-elimination settings, with few malaria cases among broader febrile illness.

**WHAT IS ALREADY KNOWN ON THIS TOPIC**

- Malaria continues to exert financial strain on households and economies in endemic settings.
- As in many other Southeast Asian settings, Indonesia offers free or heavily subsidised care for patients with malaria; however, patients often face many financial barriers to seek care.

**WHAT THIS STUDY ADDS**

- Using cross-sectional surveys as part of a *Plasmodium knowlesi* malaria surveillance study, this study compares the out-of-pocket costs and productivity losses of human-only and patients with zoonotic malaria compared with other causes of acute febrile illness, in two provinces of Indonesia.

**HOW THIS STUDY MIGHT AFFECT RESEARCH, PRACTICE OR POLICY**

- This study reveals that patients with malaria faced higher costs compared with patients with other febrile illnesses, primarily due to increased time away from usual activities.
- When designing malaria control and elimination policies, these often-hidden, indirect costs could be targeted to ensure patients with malaria have timely access to appropriate care.

## Introduction

 Malaria, caused by various parasitic protozoa of the *Plasmodium* genus, continues to cause financial and health burdens globally.^1^ Often, the greatest malaria burden occurs in individuals and households with the most limited access to healthcare,^2 3^ including financially vulnerable populations.^4^ Financial and geographical barriers can cause patients to avoid seeking adequate malaria treatment, posing a challenge for controlling and eliminating the disease.^5 6^ Six species commonly cause malaria in humans: *P. falciparum, P. vivax, P. malariae, P. ovale walikeri*, *P. ovale curtisi* and *P. knowlesi*.^7^ The Southeast Asian region has made considerable gains toward achieving malaria elimination.^1^ These efforts, however, have historically focused on controlling human-only *Plasmodium* species with the highest burden of illness, primarily *P. falciparum* and, to some extent, *P. vivax*.^1 8^ The Indonesian government has outlined the aim of eliminating malaria in all provinces by 2030^9 10^ but is not on track to meet this goal if a business-as-usual approach is continued.^11^

*P. knowlesi* is a zoonotic species with an intractable parasite reservoir in macaque hosts and is transmitted to humans by *Anopheles* leucosphyrus group mosquitos across most of Southeast Asia.^12^ *P. knowlesi* carries a high risk of severe disease (up to 9% of those presenting to health facilities with symptoms) despite predominantly lower parasite counts compared with other *Plasmodium* species.^13^,^16^ A previous study has shown that *P. knowlesi* patients in Malaysia experience similar out-of-pocket costs to patients with non-zoonotic malaria in the same setting.^17^ The emergence of *P. knowlesi* human infections across Southeast Asia poses substantial challenges to national public health control programmes due to the reduced effectiveness of conventional malaria control methods, such as insecticide-treated bed nets, with outdoor daytime biting occurring in the rural agricultural communities that are most at risk.^18^,^21^ *P. knowlesi* cases have been reported from across western and central provinces including in Sumatra and Kalimantan,^22 23^ where ongoing, lower intensity transmission of *P. vivax* and *P. falciparum* continues to occur. Quantitative evidence directly comparing zoonotic malaria, including its economic burden with human malaria and other febrile illnesses within a united framework is currently lacking in Indonesia. The emergence of *P. knowlesi* has meant these areas now have to consider control strategies that consider all types of *Plasmodium* species causing malaria to achieve elimination,^24 25^ including multisectoral One Health approaches.^26^

Malaria control in Indonesia, similar to many other Southeast Asian countries, is largely subsidised by government health insurance programmes,^6 27^ with most malaria treatment occurring within local government primary health clinics (‘Puskesmas’). Despite the high price subsidisation, Indonesian patients have been shown to face medical costs, transport, time off work and medicinal costs.^6 28^ Private health clinics exist for patients who can afford higher out-of-pocket costs^3^ and are commonly accessed due to perceived higher standard of care (including higher likelihood of seeing a doctor) and faster access to care.^29^ Measuring and quantifying household costs is important in understanding factors which drive treatment-seeking behaviours, and may be particularly relevant for hard-to-reach, remote populations.^30 31^ Considering malaria within a broader acute febrile illness (AFI) context is essential in settings with low malaria endemicity, as understanding differences in treatment-seeking and health burden could be important when evaluating current malaria clinical management and public health elimination strategies. Studies in other countries have consistently shown that in health systems with free or heavily subsidised treatment, febrile illness can result in significant indirect costs for patients and households.^32^,^34^

As areas approach malaria elimination, large economic investments are often needed for the surveillance and treatment of very few malaria cases.^35^ In these low-endemicity settings, other coexisting infectious causes of febrile illnesses grow in disease burden relevance; thus, taking into account disease aetiology and treatment-seeking behaviour context is crucial when designing malaria control and elimination strategies to ensure that malaria cases continue to receive appropriate treatment. Historically, malaria control programmes have focused on areas of high malaria transmission and disease burden;^36^ however, diagnosing and administering effective malaria treatment in areas of low endemicity is essential as these areas approach elimination. In rural Indonesia, this responsibility mainly lies with local health clinics, who receive patients with malaria alongside those with a wide range of other febrile illnesses. Customarily, cost of illness studies only consider one illness type,^37^ including malaria cost of illness studies.^6 17 28 38^ In low endemicity settings, where a small number of malaria cases occur among many patients with febrile illnesses seeking treatment, understanding the cost-of-illness in all patients with febrile illness can offer unique insights. For example, if patients with non-malarial febrile illness face high costs when seeking care, it may reduce their likelihood of seeking care when sick with malaria.^4^

The aim of this study was to estimate the total out-of-pocket costs and productivity losses of AFI episodes, including comparisons of malaria and non-malarial presentations, to patients and their households at eight rural sites in the provinces of North Sumatra and North Kalimantan in Indonesia where zoonotic malaria occurs.

## Methods

### Study design and participants

The economic questionnaires were conducted as part of a zoonotic malaria surveillance study (ZOOMAL (evaluating zoonotic malaria and agricultural and forestry land use in Indonesia))^39^ conducted between January 2022 and October 2023 at five selected health clinics in North Sumatra (representing Langkat, Dairi, Central Tapanuli and South Tapanuli districts) and three health clinics in North Kalimantan (two from Malinau district and one from Nunukan district).^40^ Eligible participants were enrolled if meeting study inclusion criteria including age >12 months, axillary temperature >37.5°C or history of fever in the last 48 hours with clinical symptoms of malaria and written informed consent was obtained (including from parent/guardian if age <18 years).

Questionnaires were administered by trained research staff to the participant (or parent/guardian if child <18 years) at two time-points: (1) in person at time of presentation to health facilities with AFI; and (2) with telephone follow-up at 7 days. The economic questionnaire collected direct and indirect household costs for each participant’s febrile episode and has been tested in other settings, including in Indonesia.^6 17^ To explore any difference in costs and treatment-seeking patterns of patients with malaria and other febrile illnesses, patients were recruited based on initial AFI status with clinically non-specific symptoms. Final malaria or non-malarial fever status was confirmed using validated ultrasensitive PCR methods for both human and zoonotic malaria on all participants for reference-standard diagnostic classification.^41^,^44^ For those with a negative PCR for malaria, other laboratory or point-of-care diagnostics targeting endemic infectious pathogens were not available in these small rural health facilities as part of standard ministry of health management to further definitively classify the aetiology of non-malarial fever. Where possible, the study team later classified the patients with non-malarial fever based on clinical presentation without laboratory testing. Patients with inconclusive PCR results (ie, positive for *Plasmodium* genus but with no specific species identified) were excluded from all analyses.

### Public and patient involvement

This study was conducted with careful consideration for patient and public involvement in North Sumatra and North Kalimantan. While patients and the public were not directly involved in the design or conduct of the research, several measures were taken to ensure culturally appropriate engagement, including data collection from local, trusted researchers and excluding potentially sensitive financial questions in the surveys.

### Data collection and valuation of productivity losses

Household out-of-pocket costs from patients with febrile illness were reported in Indonesian Rupiah, using an ingredients-based approach, whereby individual elements of the patient and caregivers’ treatment-seeking pathway were considered independently (both treatment and transport costs), then total treatment pathway summated. Total household costs were presented as the aggregate costs of direct and indirect costs for each patient with fever. Patients were asked about expenses and time away from usual activities prior to seeking care at initial presentation and followed up 7 days later to record any additional resources and time away post initial treatment.

Direct costs included all out-of-pocket expenses a patient incurs for diagnosis, clinic visits and follow-up treatments, as well as over-the-counter medication, traditional healers and transport prior to study recruitment. Cost categories were aggregated into treatment and transport for the main analysis, with transport multiplied by two to account for return travel to initial health facility. Indirect costs for patients were expressed as lost productivity, reported in the survey as days unable to do usual activities due to illness. Indirect costs were measured using a human capital approach.^45^ Days lost due to illness was the sum of self-reported time lost before attending the health centre and the number of recovery days when patients were unable to work following treatment.

Due to concerns about cultural acceptability, patients were not directly asked about income in the surveys. The value of a lost day of work was taken from the Department of Population Statistics, which uses data from the National Labour Force Survey 2022, Indonesia, where the mean monthly income was applied based on the province (North Kalimantan or North Sumatra).^46^ Monthly income was converted to daily income by assuming an average of 22 working days per month and inflated to 2023 values. In the base case analysis, children who were below the minimum age of employment (16 years) were not assigned productivity losses. Four alternate scenarios of wages and their application were also considered. The value of a single day’s work was applied to: adults who reported they were in paid income (Scenario A), to all adults using prespecified age-based wages from the Department of Population Statistics (Scenario B),^46^ and to all patients including children (Scenario C). The final scenario used estimates from Indonesia’s Integrated Statistical Service for each province’s minimum wage,^47^ which was applied to all adults in the surveyed population (Scenario D). These alternative wage estimates attempt to capture both real wages^46^ and higher, desired benchmark wages,^47^ to give insight into the economic cost of lost productivity.

### Data analysis

Patient data analysis was performed using Stata statistical software, V.14 (StataCorp LP, College Station, Texas, USA). Costs were inflated^48^ before conversion to 2023 US dollars (US$) using the World Bank exchange rate of 15 326 IDR per USD.^49^ The mean and SD were calculated for household costs. Missing data, where patients either had not completed the initial or follow-up survey, were handled by mean imputation by malaria status. An alternative scenario was considered where missing data is treated as having no cost. Mann-Whitney tests were used to identify statistically significant differences in costs between groups with two outcomes, such as sex or presence of malaria. The Kruskal-Wallis test was used to identify differences in total costs between individuals with malaria grouped by individual *Plasmodium* species.^50^

Direct treatment costs were also considered to determine catastrophic health expenditures (CHEs), which were defined as out-of-pocket expenditures exceeding thresholds of 10% and 25%^49 50^ of total 2022 provincial-specific average monthly household incomes of US$115 (North Kalimantan) and US$103 (North Sumatra).^46 51 52^

A generalised linear model (GLM) was conducted to model the marginal effects of independent variables on costs using a validated gamma-distributed dependent variable approach with a log link.^53^ Malaria status, sex, study site and age were tested against total costs. A separate model for malaria-only participants was also analysed to explore the effect of sex, study site and age on total costs for this subpopulation. Likely confounders were based on previous studies^31^ and plausibility of variables which may have impacted costs.

## Results

### Sociodemographic characteristics of study participants

Data was collected for 2244 participants across both settings, with the majority (78%; 1754) of patients from North Kalimantan (online supplemental file S3 Study Flowchart). Patient characteristics are presented in table 1. Overall, patients from the North Kalimantan sites were younger than patients in North Sumatra, with mean ages of 28.4 (SD=18.8) and 44.6 (SD=19.0), respectively. Patients most frequently stated they were in ‘unpaid work’ as their employment status, 37% and 66% from North Kalimantan and North Sumatra, respectively.

**Table 1:** Demographic and disease characteristics of study population

	North Kalimantan (n=1754)	North Sumatra (n=490)	Total (N=2244)
Sex (male), n (%)	994 (56.7)	198 (40.4)	1192 (53.1)
Age, mean (SD)	28.4 (18.8)	44.6 (19.0)	31.5 (20.0)
Age distribution, n (%)			
<5 years	168 (9.6)	3 (0.6)	171 (7.6)
5–15 years	398 (22.7)	46 (9.4)	444 (19.8)
16–35 years	560 (31.9)	105 (21.4)	665 (29.6)
36–50 years	387 (22.1)	125 (25.5)	512 (22.8)
50–65 years	200 (11.4)	138 (28.2)	338 (15.0)
>65 years	41 (2.3)	73 (14.9)	114 (5.1)
Employment status, n (%)			
Paid employment	484 (27.6)	111 (22.7)	595 (26.5)
Unpaid employment	644 (36.7)	322 (65.7)	966 (43.0)
Student	566 (32.3)	49 (10.0)	615 (27.4)
Unknown	60 (3.4)	8 (1.6)	68 (3.0)
Malaria cases, n (%)*	**137** (**7.8**)	**16** (**3.2**)	**153** (**6.8**)
*P. falciparum*	2 (1.5)	1 (6.3)	3 (2.0)
*P. knowlesi*	33 (24.1)	2 (12.5)	35 (22.9)
*P. malariae*	0 (0.0)	12 (75.0)	12 (7.8)
*P. ovale*	1 (0.7)	0 (0.0)	1 (0.7)
*P. vivax*	96 (70.1)	1 (6.5)	97 (63.4)
Mixed infection**†**	5* (3.6)	0 (0.0)	5 (3.3)
Surveys conducted, n (%)			
At initial presentation	1753 (99.9)	489 (99.8)	2242 (99.9)
At 7-day follow-up	1341 (76.5)	481 (98.2)	1822 (81.2)

Data are presented as counts, N and percentage (%) unless otherwise specified.

* Percentages for each species are expressed as a percentage of all malaria cases.

† Mixed infections were coinfections of *P. falciparum* and *P. vivax* (n=2), and coinfections of *P. knowlesi* and *P. vivax* (n=3).

*P. falciparum*, *Plasmodium falciparum*; *P. knowlesi*, *Plasmodium knowlesi*; *P. malariae*, *Plasmodium malariae*; *P. ovale*, *Plasmodium ovale*; *P. vivax*, *Plasmodium vivax*.

A total of 153 participants were confirmed to have malaria. The overall prevalence of malaria in those recruited with health economic data was higher in North Kalimantan, which had a larger number of malaria cases included in the analysis (137 cases, 7.8% prevalence) compared with North Sumatra (16 cases, 3.2% prevalence). Five species causing malaria (*P. falciparum*, *P. knowlesi*, *P. vivax, P. ovale* and *P. malariae*) were present, though no cases of *P. ovale* were diagnosed in North Sumatra and no cases of *P. malariae* were identified in North Kalimantan. Malaria severity was not able to be fully evaluated using all WHO research criteria due to the lack of routine laboratory capacity for relevant liver and renal biochemistry; however, microscopy-based parasite count quantification was evaluated for all patients, and none met the severity criterion for hyperparasitaemia.^54^ No patients died during the study.

### Disease burden

The average number of days participants spent away from usual activities was higher for those with malaria at 4.8 days (SD=8.4) total compared with 2.6 days (SD=4.5) for those with other causes of fever, respectively (p<0.001; table 2). Receiving care from family or household members either before or after presentation was almost entirely unreported in North Kalimantan regardless of malaria status, with an average of less than 0.1 days and 73% reporting no caregiving. This is likely a comprehension error rather than a true absence of caregiving. In North Sumatra, however, patients with malaria required caregiving for an average of 4.2 days (SD=8.3) compared with 0.7 days (SD=1.3) for other febrile illnesses. Total time away from usual activities for both the patient and caregiver was significantly higher for patients with malaria (5.3, SD=9.8) compared with patients with other febrile illnesses (2.8, SD=4.7; p<0.001).

**Table 2:** Mean (SD) of number of productive days lost for individual patients and households due to acute febrile illness (N=2244)

	Malaria			Non-malarial fever			P value*
	North Kalimantan (n=137)	North Sumatra (n=16)	Total (n=153)	North Kalimantan (n=1617)	North Sumatra (n=474)	Total (n=2091)	
Days away from usual activity preclinic	3.2 (3.8)	9.6 (21.8)	3.8 (8.0)	2.3 (4.7)	2.3 (2.6)	2.2 (4.3)	<0.001
Days away from usual activity postclinic	0.8 (1.4)	2.6 (2.2)	1.0 (1.6)	0.2 (0.7)	0.8 (1.4)	0.3 (0.9)	<0.001
Total days patient unable to work	**4.0** (**4.5**)	**12.2** (**21.6**)	**4.8** (**8.4**)	**2.4** (**4.8**)	**3.1** (**3.1**)	**2.6** (**4.5**)	**<0.001**
Caregiving received preclinic (days)	0.01 (0.1)	3.6 (8.1)	0.4 (2.8)	0.1 (0.4)	0.5 (0.8)	0.2 (0.6)	0.088
Caregiving received postclinic (days)	0.02 (0.1)	0.6 (1.0)	0.1 (0.4)	0.02 (0.1)	0.3 (0.8)	0.1 (0.4)	0.343
Total days of caregiving received	**0.03** (**0.1**)	**4.2** (**8.3**)	**0.5** (**2.9**)	**0.1** (**0.5**)	**0.7** (**1.3**)	**0.2** (**0.8**)	**0.776**
Total days of lost productivity	**4.0** (**4.5**)	**16.4** (**25.4**)	**5.3** (**9.8**)	**2.5** (**4.9**)	**3.8** (**3.7**)	**2.8** (**4.7**)	**<0.001**

* Mann-Whitney two sample test of all patients with malaria compared with all patients with other febrile illnesses.

### Household costs of malaria and other febrile illnesses

While patients with non-malarial febrile illness incurred a mean total cost of US$16.8 (SD=38.0) for each illness episode, this rose to US$32.9 (SD=57.0) for malaria episodes. These costs differed greatly by location, with US$25.4 (SD=29.6) in North Kalimantan much lower than the US$97.0 (SD=141.7) reported in North Sumatra (table 3). Costs were mostly comprised of indirect costs (70%) as a result of patient or caregiver productivity losses. The majority of the direct costs were due to treatment costs in North Kalimantan (55%) and North Sumatra (85%). Costs are also presented in Indonesian Rupiah in online supplemental tables 1 and 2.

**Table 3:** Total mean household costs with SD for a single febrile episode in 2023 US dollars, by location and malaria status (N=2244)

	Malaria			Non-malarial fever			P value*
	North Kalimantan (n=137)	North Sumatra (n=16)	Total (n=153)	North Kalimantan (n=1617)	North Sumatra (n=474)	Total (n=2091)	
Treatment	4.0 (10.6)	25.9 (38.3)	6.3 (17.0)	2.9 (5.3)	6.9 (28.3)	3.8 (14.3)	0.245
Transport	3.2 (7.0)	4.3 (5.1)	3.3 (6.8)	3.2 (24.4)	1.0 (2.5)	2.7 (21.5)	<0.001
Total direct costs	**7.2** (**12.3**)	**30.2** (**40.2**)	**9.6** (**18.6**)	**6.1** (**25.3**)	**7.9** (**29.4**)	**6.5** (**26.3**)	**0.002**
Patient productivity losses	17.8 (22.7)	46.4 (108.2)	20.8 (41.2)	8.0 (25.1)	12.8 (14.7)	9.1 (23.3)	<0.001
Caregiver productivity losses	0.2 (0.6)	20.3 (40.4)	2.3 (14.1)	0.5 (2.5)	3.5 (6.1)	1.2 (3.8)	0.785
Total indirect costs	**18.2** (**22.8**)	**66.8** (**125.9**)	**23.3** (**47.4**)	**8.5** (**25.3**)	**16.5** (**16.9**)	**10.3** (**23.9**)	**<0.001**
Total costs	**25.4** (**29.6**)	**97.0** (**141.7**)	**32.9** (**57.0**)	**14.6** (**37.8**)	**24.4** (**37.5**)	**16.8** (**38.0**)	**<0.001**

* Mann-Whitney two sample test of all patients with malaria compared with all patients with other febrile illness.

Consistent with the disease burden findings, patients with malaria faced higher direct (p=0.002), indirect and total costs (p<0.001 for both; table 3) compared with patients with other febrile illnesses (p<0.001 for; table 3). A further breakdown of these costs is provided in online supplemental table 3. When considering all missing data as zero costs, total costs decreased by 1% for malaria and other causes of febrile illness, respectively (online supplemental table 4). While laboratory tests were not available to further differentiate non-malarial fever, a clinical diagnosis was made using patient records for 939 of the AFIs (online supplemental table 5). For 1152, a clinical diagnosis was not possible. The indirect and total costs for each suspected clinical cause of each non-malarial fever were consistently lower than those reported for the malaria cases. For direct costs, upper respiratory tract pneumonia infections, tuberculosis and the single sepsis case had higher direct costs than the patients with malaria. These results should be interpreted with caution since they were made on the basis of record review and not confirmed by laboratory testing.

### Costs of malaria by population subgroup

Female participants reported a slightly lower mean cost of illness for malaria of US$32.1 compared with US$33.3 for males (p=0.001) (table 4). These differences were driven by higher direct costs, as indirect costs between females and males were similar.

**Table 4:** Total mean (SD) household costs and SD for a malaria episode by sex, malaria species and province in 2023 US dollars (N=153)

	n	Direct costs	Indirect costs	Total	P value
Sex					
Male	101	10.6 (16.5)	22.7 (26.5)	**33.3** (**37.3**)	0.001*
Female	52	7.5 (22.0)	24.5 (73.0)	**32.1** (**83.4**)	
Malaria species					<0.001†
*P. falciparum*	3	13.7 (13.1)	56.5 (35.4)	**70.2** (**47.8**)	
*P. knowlesi*	35	12.9 (18.9)	26.0 (26.4)	**38.9** (**37.7**)	
*P. malariae*	12	18.9 (24.8)	72.0 (145.6)	**90.9** (**161.3**)	
*P. vivax*	97	7.5 (17.9)	15.5 (20.7)	**23.0** (**31.4**)	
*P. ovale*	1	–	–	–	
Mixed infections	5	3.2 (4.1)	23.1 (27.2)	**26.3** (**30.1**)	
Location					
North Kalimantan	137	7.2 (12.3)	18.2 (22.8)	**25.4** (**29.6**)	0.006*
North Sumatra	16	30.2 (40.2)	66.8 (125.9)	**97.0** (**141.7**)	

* Mann-Whitney two sample test.

† Kruskal-Wallace t-test.

*P. falciparum*, *Plasmodium falciparum*; *P. knowlesi*, *Plasmodium knowlesi*; *P. malariae*, *Plasmodium malariae*; *P. ovale*, *Plasmodium ovale*; *P. vivax*, *Plasmodium vivax*.

Direct costs were similar for the majority of malaria cases across *Plasmodium* species, except for those with *P. vivax* infections which had lower costs, however, with increased case numbers compared with other species, high-cost patients have had less impact on mean costs (online supplemental table 3). Overall cost data were highly skewed due to high-cost outliers with high uncertainty around the mean values, particularly for the indirect costs (online supplemental table 3). The mean indirect costs were higher for *P. falciparum* and *P. malariae* episodes (US$56.5 and US$72.0, respectively) than for *P. knowlesi* (US$26.0) and *P. vivax* (US$15.5), driven by high-cost outliers and small sample sizes. The one patient with *P. ovale* did not report any direct or indirect costs. Given the small number of cases for some *Plasmodium* species, the interspecies cost comparisons should be interpreted as preliminary and descriptive.

### Scenario analyses

In the most conservative alternative scenario, where formal national wage estimates were applied only to adults who stated they were in paid employment (Scenario A), the mean cost per episode dropped from US$32.9 (base case) to US$28.2 for patients with malaria and from US$16.8 to US$12.2 for patients with non-malarial fever. When using the age-stratified wage estimates^46^ in Scenario B, the total cost per episode decreased by 2% for patients with malaria and decreased by 4% for patients with non-malarial fever (to US$31.7 and US$16.2, respectively). With the universal application of wage loss estimates to the entire patient population, Scenario C, the total costs increased by 13% and 26%, respectively, for patients with malaria and non-malarial fever, respectively. When using provincial minimum wage estimates (Scenario D), total costs increased by 78% for patients with malaria and 94% for patients with other febrile illnesses (online supplemental table 6).

**Figure 1:**
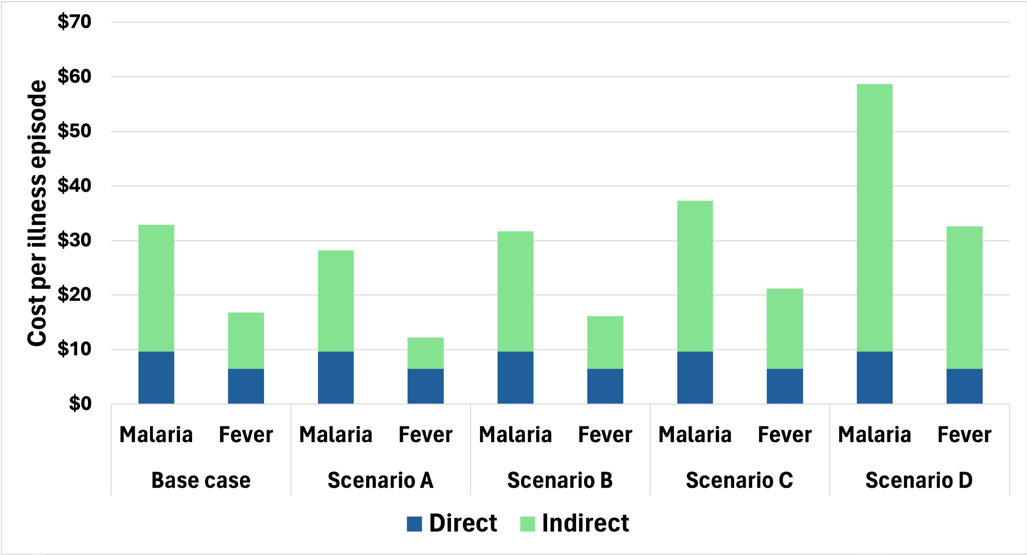
Scenario analysis of estimating the mean indirect costs and associated impact on the total cost per episode in 2022 US dollars (n=2244**)**. The base case universally applies the mean wage estimates from the Department of Population and Labour Statistics^46^ to all adults. Scenario A applies this wage only to adult patients who reported being in paid employment. Scenario B applies age-stratified wage estimates^46^ to all adults. Scenario C uses the base case wage estimate to the entire patient population, including children. Scenario D uses minimum wage estimates^47^ applied to all adults. Fever patients are considered as non-malarial fever patients.

### Catastrophic Health Expenditure

The mean direct expenditure for a single malaria episode was US$9.6. Notably, 16% of malaria cases (25/153) incurred direct costs that exceeded the 10% threshold of their provincial monthly income (US$115 (North Kalimantan) and US$103 (North Sumatra),^46^ indicating CHE for these households. For the patients with non-malarial fever, 12% (241/2091) of patients experienced CHE at the 10% monthly income threshold. Patients with malaria were more likely to experience CHE: OR 1.50 (p=0.075). When using a higher defined threshold for CHE of 25% of monthly income, this proportion decreased to 8% (12/153) of those with malaria and 3.4% (72/2091) of patients with non-malarial fever, OR 2.39 (p=0.006).

### Generalised Linear Model

The GLM tested for associations between the independent variables for sex, age, malaria status and location (North Kalimantan compared with North Sumatra) with the outcome variable of total costs (online supplemental table 7). When holding independent variables constant, malaria episodes were associated with an incremental total cost of US$11.9 (95% CI US$5.3 to US$18.4; p<0.001) compared with non-malarial fever. Across the entire population, being female was associated with a decreased total cost (−US$7.5) compared with being a male (p<0.001). Gender did not remain a statistically significant independent predictor of total cost when only considering patients with malaria in a separate regression model (online supplemental table 8). Older age was also a significant predictor of total cost, with a marginal cost of US$0.3 for each additional year of age (95% CI US$0.16 to US$0.36; p<0.001). Results of an additional GLM which only considered direct costs for all patients are presented in online supplemental table 9, where, as being female and being located in North Kalimantan were associated with a decreased total direct cost (−US$3.7; 95% CI −US$6.0 to −US$1.4); p=0.002 and −US$3.5; 95% CI −US$6.3 to −US$0.7, respectively).

## Discussion

Our study found that patients with malaria in North Kalimantan and North Sumatra face substantial financial barriers when accessing care, with a mean total cost of US$32.9 per episode, higher than the US$16.8 reported by other causes of non-malarial fever. A major finding of this study is that patients with malaria are likely to face increased time away from usual activities both before and after seeking treatment, while facing higher direct and indirect costs than those of patients with other febrile illnesses. While health clinics provide mostly subsidised care to patients,^6 27^ patients face substantial direct out-of-pocket costs, US$6.5 for patients with febrile illness rising to US$9.6 for patients with malaria, roughly 10% of patients’ monthly incomes. At both 10% and 25% thresholds, patients with malaria were significantly more likely to experience catastrophic healthcare expenditure than patients with non-malarial fever. To our knowledge, this is the first study in Indonesia that has concurrently compared the household burden of multiple *Plasmodium* species causing malaria (including zoonotic *P. knowlesi*) and other causes of AFI.

Patients with malaria and non-malarial fever in this study resided in rural and remote agricultural areas, which are inherently vulnerable to high financial and geographical barriers when seeking care. High-cost patients within this study present a challenge in malaria control, as patients who face large direct and indirect productivity costs may be less likely to seek care.^4^ Positively skewed data is common for cost data, as often a small number of patients have legitimate high expenditures that cannot be ignored.^55^ A small number of patients enrolled in this study reported the need to travel for several days, due to the extremely remote areas where malaria is present, which is particularly relevant for patients with zoonotic malaria.^4^ Although detailed transportation methods were not systematically collected within this survey, patients’ transport costs were consistent with the documented village locations in remote forests and agricultural settings, including forestry and oil palm camps. Additionally, transport costs are likely underestimated in this study, as return travel expenses were not directly captured.

These findings demonstrate how it can often be prohibitively costly, and therefore unfeasible, to travel to healthcare centres to receive appropriate treatment for malaria and other febrile illnesses^4^; hence, patients forego care or seek inappropriate care from other sources.^56 57^ Considering Indonesia’s ambitious malaria elimination targets,^9 10^ ensuring the capacity for timely malaria diagnosis and treatment availability in remote and hard-to-reach populations will continue to be important when evaluating the public health control policies which have been successful in reducing the prevalence of *P. vivax* and *P. falciparum* in Sumatra and Kalimantan.^9 10 58^ Policymakers could consider household level subsidies,^59^ or subsidised, outreach-based test-and-treat strategies^60^ which have shown to be effective in other settings.

The recent emergence of zoonotic *P. knowlesi* now means these settings are treating patients with both human-only and zoonotic malaria.^61^ The Indonesian National Malaria Control Programme is developing appropriate surveillance activities, including centralised laboratory PCR confirmation for suspected *P. knowlesi* cases, and inclusion in the national electronic reporting system, to accurately evaluate the threat and distribution of emerging zoonotic malaria. Findings on the costs associated with zoonotic malaria will provide further evidence on the impacts of increasing incidence and the clinical spectrum of disease related to *P. knowlesi* malaria in endemic areas of western Indonesia. This study indicated that *P. knowlesi* cases had similar costs to other species of malaria, and thus higher caseloads of zoonotic malaria increase the overall economic burden of illness. The ability for diagnostic tools to differentiate *P. knowlesi* infections from other species of *Plasmodium* will be crucial^62^ in Indonesia and co-endemic settings in Southeast Asia. This is highly relevant for areas in Kalimantan, including Ibu Kota Nusantara where Indonesia is currently relocating their capital city, with forest fragmentation due to logging activities and conversion to oil palm plantations resulting in increasing contact between humans, vectors and macaque hosts and higher zoonotic malaria acquisition risk.^63^

Our study found *P. vivax* patients experienced lower out-of-pocket costs than other *Plasmodium* species, which contrasts with previous studies in the region.^17^ While difficult to ascertain given data availability and understanding, this may be a result of varying requirements for follow-up of primaquine treatment, with other endemic countries mandating follow-up for the full duration of primaquine treatment. Our study also showed lower costs for females; however, this may be due to sociocultural factors.^64^ It is worth noting that in the regression model restricted to patients with malaria, sex was not a significant predictor of total costs (online supplemental table 8), suggesting that the observed difference should be interpreted with caution. Older age was also associated with higher costs, important when considering that *P. knowlesi* malaria infections often occur in older age groups. Household costs showed a consistent trend with those for both malaria and non-malarial fever, comprising mainly productivity losses, rather than direct expenditure. While laboratory diagnosis for other causes of AFI was not routinely available, evaluation of the provisional clinical diagnoses was consistent with malaria having higher costs than non-malarial fever. However, due to the lack of laboratory confirmation, these results should be interpreted with caution. While this is a study limitation, it also reflects the real-world clinical context in primary healthcare settings where febrile illnesses are managed without definitive aetiological information. The finding that malaria resulted in higher costs remained compelling across all analyses.

Overall, our findings emphasise the public health importance of patients with malaria avoiding additional personal financial disincentives when seeking treatment.^5^ Including productivity losses in economic analyses is helpful to understand the true economic impact of an illness,^65^ particularly in low- and middle-income settings, where time away from economic activity is often unfeasible and detrimental to household financial security.^66 67^ Considering a more holistic approach to economic analyses where productivity losses are valued can alter the interpretation of economic evidence, providing more complete evidence to achieve efficient allocation of resources.^66 68^ Additionally, the findings on CHE showed the relative impact of direct malarial illness costs on household financial stability. However, as productivity losses were the major component of total costs, this metric likely does not entirely capture the impact of malarial illness on household financial stability for this population.

The application of a variety of wage estimate scenarios in this study demonstrated important differences in quantifying productivity losses, consistently indicating higher costs for patients with malaria compared with patients with non-malarial fever. Scenarios A, B and C, applying mean estimates to only paid workers, the entire population and using age-specific wages, remained reasonably consistent to the base case estimates for both malaria and non-malarial fever. However, when using the provincial minimum wage estimates from the Integrated Statistical Service^47^ as an alternative higher wage estimate overall, total costs increased 78% for patients with malaria, from baseline to US$58.7 (online supplemental table 6). These minimum wage estimates are likely to overestimate true wages in these settings, as they are recommendations of minimum wage based on provincial gross domestic product, which can distort the true wages of workers in these provinces. This is particularly true for the rural settings where malaria tends to be found. Regardless of the method used, productivity losses of patients with malaria were substantial. Thus, preventing malaria cases offers financial protection to these populations not only through saved direct spending from treatment seeking, but retaining economic output from usual activities.

Our study had several additional limitations. First, the questionnaire did not include questions about the incomes of patients or caregivers. Thus, we have valued productivity losses with secondary sources, which may not accurately reflect the incomes of the study populations. However, both the Department of Population Statistics^46^ and the Integrated Statistical Service^47^ are widely used government estimates, and the mean income for the former was chosen for the base case analysis because it provided provincial level data. In addition, the study questionnaire did not ask about the mode of transportation to access health facilities, which would have aided interpretation for high-cost patients. Additionally, the small case numbers of *P. falciparum* and *P. malariae* mean that the cost comparisons between species are limited in usefulness. Specifically, one *P. malariae* patient reported 90 days away from usual activities which heavily influenced the mean costs for this species. When removing this patient from the analysis, there was a substantial drop in mean costs for *P. malariae* species, but not the mean cost for all malaria cases. Caregiving was almost entirely unreported in North Kalimantan study sites (table 2), including for patients under 5 years. These findings indicate that the question may have been misunderstood by caregivers, leading to the underestimation of productivity losses and therefore total costs. Patients in North Sumatra also reported taking more time off usual activities due to illness (12 days as compared with 4 in North Kalimantan, (online supplemental table 10). These differences in indirect costs could indicate cultural differences across provinces in terms of how illnesses are perceived or coped with. Future qualitative research could help explain why this was an issue and explore how to ask these questions in the future. The sites did not only report differences in indirect costs, but also in direct costs due to malaria illness. The increased direct costs in North Sumatra could be due to treatment at public health centres as compared with the government hospital where most participants in North Kalimantan were recruited from as well as differences in treatment-seeking before study enrolment. Due to the specific study locations, the generalisability of these findings to other regions in Indonesia may be limited, particularly given the small number of malaria cases present in Sumatra.

Another key limitation of our study was the inability to logistically contact some patients via telephone for the follow-up cost surveys. The potential bias from our use of mean imputation by malaria status in the base case estimate^69^ was likely minimal as demonstrated by our validation analyses where missing data were given zero costs with no meaningful difference from the base case (online supplemental table 4). Furthermore, the majority of the costs per AFI episode (>90%) were reported at recruitment for the 81% of patients who completed both surveys. Without further data of these participants’ context, it is difficult to determine the randomness of their missing data.

Finally, the clinical diagnosis of other febrile illnesses was not available, which meant that likely diverse infectious and potentially non-infectious causes of AFI were to be analysed as a distinct group. Future research could try to stratify these illnesses in more detail for more robust comparisons, although the higher costs for malaria compared with this large heterogeneous alternative febrile illness group were consistent across different analyses and productivity loss scenarios.

## Conclusion

To our knowledge, this is the first study to estimate the economic burden to Indonesian households of zoonotic and human-only malaria, alongside non-malarial febrile illness. Patients with malaria experienced substantial out-of-pocket costs despite highly subsidised treatment, which could disincentivise patients from seeking adequate care. Patients with malaria consistently had longer illness duration and higher direct costs than patients with non-malarial febrile illness in the same settings. Direct costs and productivity losses could impact households experiencing disadvantage and cause catastrophic economic impacts for these hard-to-reach communities. Given the considerable indirect productivity losses consistently found in these patient populations, policies such as short-term economic compensation could be a viable option in these settings. As areas nearing malaria elimination face the challenges of ongoing non-zoonotic malaria susceptible populations and the emergence of zoonotic malaria transmission to fill this vacant ecological niche, it becomes more important to remove any practical or financial barriers to patients receiving effective malaria treatment. Zoonotic malaria now jeopardises elimination efforts in areas of western Indonesia, with substantial patient cost implications justifying the need to strengthen control efforts for both zoonotic and human-only *Plasmodium* species infections.

## Supplementary material

10.1136/bmjgh-2025-020504 online supplemental file 1

10.1136/bmjgh-2025-020504 online supplemental table 1

10.1136/bmjgh-2025-020504 online supplemental file 2

## Data Availability

Data are available in a public, open access repository. Available: https://github.com/PatrickAbraham1/ZooMAL-Costing.^70^
