# Supplementary material for: The household economic burden of human-only and zoonotic malaria, compared to other causes of acute febrile illness in Indonesia
Source: BMJ Glob Health. 2026 Mar 26;11(3):e020504. doi: 10.1136/bmjgh-2025-020504 (PMC13034341; doi:10.1136/bmjgh-2025-020504)
Supplement: online supplemental file 1 [file bmjgh-11-3-s001.docx]

# **Appendix – Reflexivity Statement**

**How does this study address local research and policy priorities?**

Our study, “The larger household economic burden of human-only and zoonotic malaria, compared to other causes of acute febrile illness in Indonesia,” is part of the larger ZOOMAL project (Evaluating ZOOnotic MALaria transmission and agricultural land use in Indonesia). This project has been codeveloped and implemented with Indonesian research teams to strengthen surveillance of zoonotic malaria in Indonesia and inform public health control efforts and sustainable agricultural development. This research aims to inform Indonesian policy on the best ways to conduct surveillance and control efforts to combat the threat of zoonotic *Plasmodium knowlesi*.

**How were local researchers involved in study design?**

Local researchers were integral in the design and implementation of the ZOOMAL. This research was undertaken from conceptualisation to analysis in partnership with The Eijkman Institute for Molecular Biology (Indonesia) and the Universitas Sumatera Utara (Indonesia). Authors INL, RN, and HS are local researchers who designed and ran the entire ZOOMAL study, including designing the economic burden components with MJG and AD.

**How has funding been used to support the local research team?**

Funding was used to support the local research teams through direct salary support of fieldworkers, laboratory staff and local principal investigators, as well as laboratory equipment and consumables.

**How are research staff who conducted data collection acknowledged?**

Research staff who were involved in the study are acknowledged in the “Acknowledgements” section. “We thank the study participants, the Indonesian Research Teams based at Universitas Sumatera Utara, Provincial Health Office of North Sumatra, Provincial Health Office of North Kalimantan, Exeins Health Initiative, Malinau Region Public Hospital, Malinau and Nunukan District Health Offices, and the National Malaria Control Programme, Indonesian Ministry of Health.”

**Do all members of the research partnership have access to study data?**

All research team members have access to study data, which has been made available via an online repository.

**How was data used to develop analytical skills within the partnership?**

Research institutions involved in the study (USU and EHI) were trained to have the skills and knowledge to use accurate molecular detection methods for zoonotic malaria surveillance. PA was responsible for data analysis and was supervised by AD and MJG throughout the analysis phases.

**How have research partners collaborated in interpreting study data?**

This research has been built on a continued relationship between Indonesian and Australian research institutes. Monthly research meetings and presentations of data and results were consistently shared between research partners in online meetings and with all study teams in a workshop in Jakarta, Indonesia. Data clarifications and interpretations have been an iterative process between all research partners.

**How were the research partners supported to develop writing skills?**

The research team writing this manuscript was led by an early career researcher (PA) with consistent supervision from senior academics MJG, AD, NA, and feedback from lead investigators INL and RN. Writing supervision was also given by senior academic HS.

**How will research products be shared to address the local needs?**

This manuscript will be made open-access once published. This research will also be shared internally with study teams and with the respective Indonesian Government Ministries by local researchers.

**How is the leadership, contribution and ownership of this work by LMIC researchers recognised within the authorship?**

Most of the authorship team consists of local researchers (nine out of thirteen authors). This includes lead investigators from Indonesian research institutes (INL and RN), who are all responsible for data management and supervision.

**How have early career researchers across the partnership been included within the authorship team?**

Early career researchers have featured heavily in the authorship team, including PA as 1^st^ author. Early career researchers make up the majority of authors (9 out of 15).

**How has gender balance been addressed within the authorship?**

The authorship team consists of eight females and seven males. The ZOOMAL Project has been strongly committed to ensuring equitable gender outcomes through training, building leadership and research skills and empowerment. Over half of the broader project team are women (26 of 48 people) who are incredible role models for our project members and a wide range of collaborators, networks, and stakeholders.

**How has the project contributed to the training of LMIC researchers?**Through the project, researchers from low- and middle-income settings have benefited from extensive capacity building in training, leadership development, and research skill enhancement. This included conferences, workshops, and short courses such as a One Health Summer Course and specialised malaria surveillance and entomology training. Local researchers gained hands-on experience in field research, molecular techniques, and data analysis tools. These efforts have strengthened individual and institutional capabilities and laid a foundation for improved regional zoonotic disease prevention and public health surveillance.

**How has the project contributed to improvements in local infrastructure?**

This project has aided in developing gold-standard detection methods to evaluate zoonotic and human-only malaria disease burden.

**What safeguarding procedures were used to protect local study participants and researchers?**

All data was deidentified as part of data collection, including potentially sensitive clinical and financial data. No data on patient’s incomes were collected during the study, as this was not deemed appropriate within these settings. The study strictly adhered to ethics approvals stated within the manuscript. “This study was approved by institutional review boards in Indonesia, including the Universitas Sumatera Utara (NO:723/KEP/USU/2021), the Eijkman Institute of Molecular Biology (#169), and the Human Research Ethics Committee of Menzies School of Health Research, Australia (HREC#22-4417). All adult participants provided written informed consent, with parental/guardian written informed consent gained for participants less than 18 years of age.”
