# Supplementary material for: The household economic burden of human-only and zoonotic malaria, compared to other causes of acute febrile illness in Indonesia
Source: BMJ Glob Health. 2026 Mar 26;11(3):e020504. doi: 10.1136/bmjgh-2025-020504 (PMC13034341; doi:10.1136/bmjgh-2025-020504)
Supplement: online supplemental table 1 [file bmjgh-11-3-s002.docx]

# **SUPPLEMENTARY MATERIALS**

**Table of contents**

Supplementary Table 1. Total mean household costs and standard deviations for a single febrile episode in 2023 Indonesian Rupiah

Supplementary Table 2. Total mean household costs with standard deviations for a malaria episode by sex and species in 2023 Indonesian Rupiah

Supplementary Table 3. Total mean, standard deviation, median, and interquartile ranges for household costs for a single malaria episode in North Kalimantan and North Sumatra

Supplementary Table 4. Total mean and standard deviation of household costs for a single febrile episode considering missing values as no cost

Supplementary Table 5. Total mean household costs with standard deviations for laboratory-confirmed malaria diagnoses and suspected clinical diagnoses

Supplementary Table 6. Total mean household costs with standard deviations for a single febrile episode using provincial minimum wage estimates.

Supplementary Table 7. Marginal costs of factors associated with variability in total household costs from the Generalised Linear Model for the entire population.

Supplementary Table 8. Marginal costs of factors associated with variability with total household costs from the Generalised Linear Model of malaria patients.

Supplementary Table 9. Marginal costs, standard errors and 95% confidence intervals of factors associated with variability with total direct costs from the Generalised Linear Model.

Supplementary Table 10. Mean and standard deviations of number of productive days lost for individual patients and households due to acute febrile illness.

### **Supplementary Table 1.** Total mean household costs and standard deviations for a single febrile episode in 2023 Indonesian Rupiah, by location and malaria status (N=2,244).

|  | Malaria (n=153) | | | Non-malarial fever (n=2,091) | | |
| --- | --- | --- | --- | --- | --- | --- |
|  | **North Kalimantan (n=137)** | **North Sumatra  (n=16)** | **All malaria  (n=153)** | **North Kalimantan (n=1,617)** | **North Sumatra (n=474)** | **All fever**  **(n=2,091)** |
| Treatment | 60,900 (161,000) | 395,000 (583,000) | 96,000 (259,000) | 45,000 (80,000) | 106,000 (431,000) | 59,000 (218,000) |
| Transport | 48,000 (107,000) | 65,000 (65,000) | 50,000 (104,000) | 48,000 (372,000) | 15,000 (38,000) | 41,000 (328,000) |
| Total direct costs | 109,000 (187,000) | 460,000 (613,000) | 146,000 (283,000) | 93,000 (386,000) | 121,000 (449,000) | 99,000 (401,000) |
| Time off work | 271,000 (346,000) | 708,000 (1,649,000) | 317,000 (627,000) | 122,000 (383,000) | 196,000 (224,000) | 139,000 (355,000) |
| Caregiver productivity losses | 3,000 (9,000) | 310,000 (616,000) | 35,000 (215,000) | 7,000 (37,000) | 54,000 (93,000) | 18,000 (58,000) |
| Total indirect costs | 274,000 (345,000) | 1,018,000 (1,919,000) | 352,000 (723,000) | 129,000 (385,000) | 250,000 (258,000) | 156,000 (363,000) |
| Total costs | 383,000 (449,000) | 1,478,000 (2,159,000) | 497,000 (868,000) | 222,000 (576,000) | 370,000 (571,000) | 256,000 (578,000) |

**Supplementary Table 2.** Total mean household costs with standard deviations for a malaria episode by sex and species in 2023 Indonesian Rupiah (N=153).

|  | *n* | Direct costs | Indirect costs | Total | P-value |
| --- | --- | --- | --- | --- | --- |
| Sex | | | | | |
| Male | 101 | 162,000 (252,000) | 341,000 (404,000) | 503,000 (567,000) | 0.001* |
| Female | 52 | 114,000 (336,000) | 373,000 (1,112,000) | 487,000 (1,271,000) |  |
| Malaria species | |  |  |  |  |
| *P. falciparum* | 3 | 209,000 (200,000) | 853,000 (545,000) | 1,063,000 (732,000) | <0.001** |
| *P. knowlesi* | 35 | 196,000 (289,000) | 393,000 (401,000) | 589,000 (572,000) |  |
| *P. malariae* | 12 | 288,000 (377,000) | 1,098,000 (2,219,000) | 1,386,000 (2,457,000) |  |
| *P. ovale* | 1 | - | - | - |  |
| *P. vivax* | 97 | 114,000 (273,000) | 232,000 (314,000) | 347,000 (477,000) |  |
| Mixed infections | 5 | 48,000 (62,000) | 348,000 (415,000) | 397,000 (465,000) |  |
| Location | |  |  |  |  |
| Kalimantan | 137 | 109,000 (187,000) | 274,000 (345,000) | 383,000 (449,000) | 0.006* |
| Sumatra | 16 | 460,000 (613,000) | 1,018,000 (1,919,000) | 1,478,000 (2,159,000) |  |

* Mann-Whitney two sample test

** Kruskal Wallace t test

**Supplementary Table 3.** Total mean, standard deviation (SD), median, and interquartile ranges (IQR) for household costs for a single malaria episode in North Kalimantan and North Sumatra in 2023 United States dollars (N=2,244).

|  | Mean | SD | Median | IQR | Mean | SD | Median | IQR |
| --- | --- | --- | --- | --- | --- | --- | --- | --- |
| North Kalimantan (N=1,754) | **Malaria (n=137)** | | | | **Non-malarial fever (n=1,617)** | | | |
| Treatment | 4.0 | 10.6 | 0.02 | 0.0 – 5.3 | 2.9 | 5.3 | 0.4 | 0.0 – 5.3 |
| Travel to clinic or hospital | 3.2 | 7.0 | 1.2 | 0.9 – 2.6 | 3.2 | 24.4 | 1.1 | 0.7 – 2.0 |
| Total direct costs | 7.2 | 12.3 | 4.5 | 0.9 – 7.4 | 6.1 | 25.3 | 2.6 | 0.7 – 7.1 |
| Patient productivity losses | 17.8 | 22.7 | 10.9 | 0.0 – 27.0 | 8.0 | 25.1 | 0 | 0.0 – 10.9 |
| Caregiver productivity losses | 0.2 | 0.6 | 0.0 | 0.0 – 0.0 | 0.5 | 2.4 | 0.0 | 0.0 – 0.0 |
| Total indirect costs | 18.2 | 22.8 | 10.9 | 0.0 – 27.2 | 8.5 | 25.3 | 2.3 | 0.0 – 10.9 |
| Total costs | 25.4 | 29.6 | 17.1 | 4.3 – 33.5 | 14.6 | 37.8 | 8.2 | 1.6 – 18.1 |
|  |  |  |  |  |  |  |  |  |
| North Sumatra (N=490) | **Malaria (n=16)** | | | | **Non-malarial fever (n=474)** | | | |
| Treatment costs | 25.9 | 38.3 | 6.0 | 1.8 – 48.8 | 6.9 | 28.3 | 2.3 | 0.0 – 4.3 |
| Travel costs to clinic or hospital | 4.3 | 5.1 | 2.2 | 1.3 – 5.2 | 1.0 | 2.5 | 0.7 | 0.0 – 1.3 |
| Total direct costs | 30.2 | 40.2 | 7.5 | 3.3 – 53.9 | 7.9 | 29.4 | 2.7 | 0.7 – 5.3 |
| Patient productivity losses | 46.4 | 108.2 | 21.9 | 0.0 – 29.1 | 12.8 | 14.7 | 9.7 | 0.0 – 19.4 |
| Caregiver productivity losses | 20.3 | 40.4 | 0.0 | 0.0 – 14.6 | 3.5 | 6.1 | 0.0 | 0.0 – 7.3 |
| Total indirect costs | 66.8 | 125.9 | 29.1 | 7.3 – 58.3 | 16.5 | 16.9 | 14.6 | 4.9 – 23.1 |
| Total costs | 97.0 | 141.7 | 34.0 | 11.5 – 114.3 | 24.4 | 37.5 | 17.2 | 8.3 – 29.1 |

### **Supplementary Table 4.** Total mean and standard deviation of household costs for a single febrile episode considering missing values as no cost.* Costs are by location and malaria status in 2023 United States Dollars (N=2,244).

|  | Malaria (n=153) | | | Non-malarial fever (n=2,091) | | |
| --- | --- | --- | --- | --- | --- | --- |
|  | **North Kalimantan (n=137)** | **North Sumatra  (n=16)** | **Overall (n=153)** | **North Kalimantan (n=1,617)** | **North Sumatra (n=474)** | **Overall**  **(n=2,091)** |
| Treatment | 3.8 (10.5) | 25.9 (38.3) | 6.1 (17.0) | 2.9 (5.3) | 6.9 (28.3) | 3.8 (14.3) |
| Transport | 3.2 (7.0) | 4.3 (5.1) | 3.3 (6.8) | 3.2 (24.4) | 1.0 (2.5) | 2.7 (21.5) |
| Total direct costs | 7.0 (12.2) | 30.2 (40.2) | 9.4 (18.5) | 6.0 (25.3) | 7.9 (21.5) | 6.5 (26.3) |
| Patient productivity losses | 16.2 (22.3) | 46.4 (108.2) | 20.0 (41.0) | 7.8 (25.1) | 12.9 (14.7) | 8.9 (23.3) |
| Caregiver productivity losses | 0.1 (0.6) | 20.3 (40.4) | 2.2 (14.1) | 0.4 (2.4) | 3.5 (6.1) | 1.1 (3.9) |
| Total indirect costs | 17.0 (22.2) | 66.8 (125.9) | 22.2 (47.3) | 8.1 (25.2) | 16.4 (16.9) | 10.0 (23.8) |
| Total costs | 25.0 (29.4) | 97.0 (141.7) | 32.5 (57.0) | 14.5 (37.8) | 24.3 (37.5) | 16.7 (37.9) |

*In those lost to follow up at day 7 the proportion for the key indicator of malaria status did not differ, nor did was median age substantially different (34 vs 31 years). There was a slightly higher proportion of males lost to day 7 follow up.

**Supplementary Table 5.** Total mean household costs with standard deviations (where possible) for laboratory-confirmed malaria diagnoses (N=153) and suspected clinical diagnoses post treatment (N=939).

| Diagnosis | n | Direct costs | Indirect costs | Total costs |
| --- | --- | --- | --- | --- |
| Malaria | 153 | 9.6 (18.6) | 23.3 (47.4) | 32.9 (57.0) |
| Suspected clinical diagnosis (n=939) |  |  |  |  |
| Dengue | 557 | 6.6 (8.4) | 10.2 (12.1) | 16.8 (15.4) |
| Typhoid | 140 | 8.8 (15.4) | 21.7 (66.3) | 30.4 (70.8) |
| Upper-respiratory tract pneumonia infection | 116 | 11.0 (25.0) | 16.1 (15.6) | 27.1 (37.2) |
| Influenza | 115 | 7.9 (46.7) | 15.1 (10.3) | 23.2 (48.6) |
| Tuberculosis | 7 | 10.4 (12.0) | 11.7 (9.3) | 22.1 (18.1) |
| Rickettsia | 2 | 1.3 | 0 | 1.3 |
| Leptospirosis | 1 | 0.7 | 10.9 | 11.5 |
| Sepsis | 1 | 22.0 | 5.4 | 27.5 |
| Undifferentiated fever | 1,152 | 5.7 (29.0) | 7.3 (16.6) | 13.0 (35.8) |

**Supplementary Table 6.** Total mean household costs with standard deviations for a single febrile episode in 2023 United States dollars, by location and malaria status (N=2,244) using provincial minimum wage estimates.

|  | Malaria (n=153) | | | Non-malarial fever (n=2,091) | | | P value* |
| --- | --- | --- | --- | --- | --- | --- | --- |
|  | **North Kalimantan (n=137)** | **North Sumatra  (n=16)** | **All malaria  (n=153)** | **North Kalimantan (n=1617)** | **North Sumatra (n=474)** | **All fever**  **(n=2091)** |  |
| Treatment | 4.0 (10.6) | 25.9 (38.3) | 6.3 (17.0) | 2.9 (5.3) | 6.9 (28.3) | 3.8 (14.3) | 0.245 |
| Transport | 3.2 (7.0) | 4.3 (5.1) | 3.3 (6.8) | 3.2 (24.4) | 1.0 (2.5) | 2.7 (21.5) | 0.001 |
| Total direct costs | 7.2 (12.3) | 30.2 (40.2) | 9.6 (18.6) | 6.1 (25.3) | 7.9 (29.4) | 6.5 (26.3) | 0.002 |
| Patient productivity losses | 39.5 (44.7) | 95.8 (169.7) | 45.4 (70.2) | 24.1 (48.3) | 24.3 (24.2) | 24.2 (44.0) | <0.001 |
| Caregiver productivity losses | 0.3 (1.1) | 32.9 (65.4) | 3.7 (22.9) | 0.8 (4.5) | 5.7 (9.9) | 1.9 (6.5) | 0.784 |
| Total indirect costs | 39.8 (44.6) | 128.8 (199.4) | 49.1 (80.3) | 25.0 (48.6) | 30.0 (29.0) | 26.1 (44.9) | <0.001 |
| Total costs | 46.9 (59.2) | 159.0 (211.4) | 58.7 (88.4) | 31.1 (57.5) | 38.0 (46.8) | 32.6 (55.3) | <0.001 |

*Mann-Whitney two sample test of all malaria patients compared to all other febrile patients

**Supplementary Table 7.** Marginal costs (in 2023 United States dollars), standard errors, and 95% confidence intervals of factors associated with variability in total household costs from the generalised linear model for the entire population (n=2,244).

|  | Marginal cost from base case (dy/dx) | Standard error | P-value | 95% confidence interval |
| --- | --- | --- | --- | --- |
| Sex (Female) | -7.47 | 1.73 | <0.001 | -10.86, -4.07 |
| Age | 0.25 | 0.05 | <0.001 | 0.16, 0.36 |
| Malaria | 11.86 | 3.32 | <0.001 | 5.34, 18.37 |
| Study site | -9.47 | 2.16 | <0.001 | -13.72, -5.23 |

**Supplementary Table 8.** Marginal costs, standard errors and 95% confidence intervals of factors associated with variability with total household costs from the Generalised Linear Model of malaria patients (N=153).

|  | Marginal Cost from base case (dy/dx) | Standard error | P-value | 95% confidence interval |
| --- | --- | --- | --- | --- |
| Sex (Female) | -14.7 | 8.6 | 0.088 | -31.5, 2.2 |
| Age | 1.15 | 0.4 | 0.005 | 0.35, 2.0 |
| Location* | -57.1 | 18.0 | 0.001 | -92.4, -21.9 |

*Marginal effect of being a female compared to male
**Marginal effect of being in Kalimantan compared to Sumatera

**Supplementary Table 9.** Marginal costs, standard errors and 95% confidence intervals of factors associated with variability with total direct costs from the Generalised Linear Model (N=2,244).

|  | Marginal Cost from base case (dy/dx) | Standard error | P-value | 95% confidence interval |
| --- | --- | --- | --- | --- |
| Sex (Female) | -3.7 | 1.2 | 0.002 | -6.0, -1.4 |
| Age* | -0.04 | 0.03 | 0.224 | -0.09, 0.02 |
| Malaria | 2.0 | 2.1 | 0.338 | -2.1, 6.1 |
| Location** | -3.5 | 1.4 | 0.013 | -6.3, -0.7 |

*Marginal effect of being a female compared to male
**Marginal effect of being in Kalimantan compared to Sumatra

**Supplementary Table 10.** Mean and standard deviations of number of productive days lost for individual patients and households due to acute febrile illness (N=2,244).

|  | North Kalimantan | | | North Sumatra | | |
| --- | --- | --- | --- | --- | --- | --- |
|  | **Malaria (n=137)** | **Non-malarial fever (n=1,617)** | **P value** | **Malaria (n=16)** | **Non-malarial fever (n=474)** | **P value** |
| Days away from usual activity pre-clinic | 3.2 (3.8) | 2.3 (4.7) | 0.001 | 9.6 (21.8) | 2.3 (2.6) | 0.010 |
| Days away from usual activity post-clinic | 0.8 (1.4) | 0.2 (0.7) | <0.001 | 2.6 (2.2) | 0.8 (1.4) | <0.001 |
| Total days patient unable to work | 4.0 (4.5) | 2.4 (4.8) | <0.001 | 12.2 (21.6) | 3.1 (3.1) | <0.001 |
| Caregiving received pre-clinic (days) | 0.01 (0.1) | 0.1 (0.4) | 0.113 | 3.6 (8.1) | 0.5 (0.8) | 0.211 |
| Caregiving received post-clinic (days) | 0.02 (0.1) | 0.02 (0.1) | 0.395 | 0.6 (1.0) | 0.3 (0.8) | 0.111 |
| Total days of caregiving received | 0.03 (0.1) | 0.1 (0.5) | 0.973 | 4.2 (8.3) | 0.7 (1.3) | 0.191 |
| Total days of lost productivity | 4.0 (4.5) | 2.5 (4.9) | <0.001 | 16.4 (25.4) | 3.8 (3.7) | <0.001 |
