# Supplementary figures and images for: The household economic burden of human-only and zoonotic malaria, compared to other causes of acute febrile illness in Indonesia
Source: BMJ Glob Health. 2026 Mar 26;11(3):e020504. doi: 10.1136/bmjgh-2025-020504 (PMC13034341; doi:10.1136/bmjgh-2025-020504)

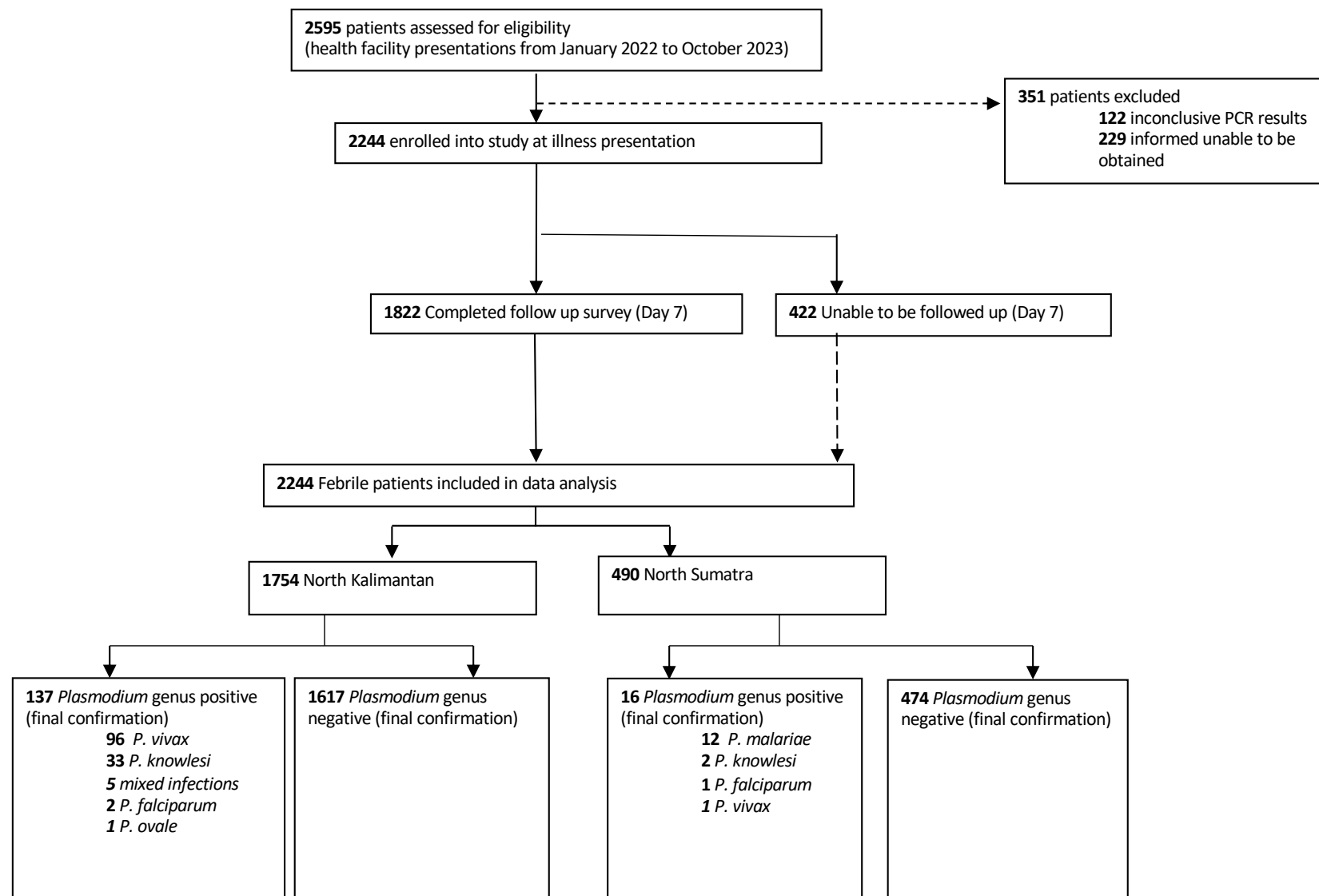

Supplement: online supplemental file 2 [file bmjgh-11-3-s003.pdf]
